# Using data from the 100,000 Genomes Project to resolve conflicting interpretations of a recurrent *TUBB2A* mutation

**DOI:** 10.1136/jmedgenet-2020-107528

**Published:** 2021-02-05

**Authors:** Vassilis Ragoussis, Alistair T Pagnamenta, Rebecca L Haines, Edoardo Giacopuzzi, Martin A McClatchey, Julian R Sampson, Mohnish Suri, Alice Gardham, Jan-Maarten Cobben, Deborah Osio, Andrew E Fry, Genomics England Research Consortium, Jenny C Taylor

**Affiliations:** 1 Wellcome Centre for Human Genetics, Oxford University, Oxford, Oxfordshire, UK; 2 NIHR Biomedical Research Centre, Oxford, UK; 3 East Midlands Regional Molecular Genetics Service, Nottingham University Hospitals NHS Trust, Nottingham, UK; 4 Institute of Medical Genetics, University Hospital of Wales, Cardiff, UK; 5 Division of Cancer and Genetics, School of Medicine, Cardiff University, Cardiff, UK; 6 Nottingham Clinical Genetics Service, Nottingham University Hospitals NHS Trust, Nottingham, UK; 7 North West Thames Regional Genetics Service, Northwick Park Hospital, Harrow, London, UK; 8 Department of Pediatrics, Amsterdam University Medical Centres, Duivendrecht, Noord-Holland, Netherlands; 9 West Midlands Regional Clinical Genetics Service and Birmingham Health Partners, Birmingham Women’s and Children’s Hospitals NHS Foundation Trust, Birmingham, UK

**Keywords:** central nervous system diseases, epilepsy, genetic techniques, germ-line mutation, sequence alignment

Defects in tubulin beta 2A class IIa (*TUBB2A*) are associated with a range of complex cerebral cortex dysplasias.^1^ Despite several studies reporting NM_001069.3:c.743C>T p.(Ala248Val) as a recurrent pathogenic mutation,^1 2^ it is listed in ClinVar with conflicting interpretations. To resolve these inconsistencies, we scanned data from the 100,000 Genomes Project^3^ (100KGP) and identified 58 individuals where p.(Ala248Val) had been called. Read alignment analysis suggested that the variant was genuine in 5/58 individuals, all of whom had a primary neurodevelopmental phenotype. In the remaining cases which spanned non-specific disease phenotypes, low allelic ratios (1%–19%) suggest recurrent mismapping artefacts.

Alpha and beta tubulins form heterodimers that polymerise to form microtubules, dynamic components of the cytoskeleton that play an important role in cell division, migration and intracellular transport. Variants in several tubulin genes are associated with a variety of cortical brain malformation phenotypes, including lissencephaly, polymicrogyria, microlissencephaly and simplified gyration, collectively termed ‘tubulinopathies’.^4 5^ A recently described tubulinopathy involving *TUBB2A* (MIM #615763) has been associated with brain phenotypes ranging from a normal cortex to extensive dysgyria.^2^ One particular *TUBB2A* variant, p.(Ala248Val), has been reported in several studies, in most cases arising de novo.^1 2 6–8^ Additional unpublished clinical cases also report a de novo origin (www.ncbi.nlm.nih.gov/clinvar/variation/127101).

Multiple occurrences of the same de novo mutation in patients with overlapping phenotypes would typically provide strong evidence supporting pathogenicity. However, on closer inspection, p.(Ala248Val) becomes harder to interpret, particularly when applying the American College of Medical Genetics and Genomics (ACMG) population allele frequency (AF) criteria PM2/BS1.^9^ The AF in gnomAD v2.1.1 is 8/237 044 in exomes and 79/15 882 in genomes, an unexpected skew for a coding variant. In gnomAD v3.1, the global AF of 400/111 804 rises to 342/24 540 (1.4%) in Africans, well above the normal threshold for a highly penetrant autosomal–dominant condition.

The p.(Ala248Val) variant fails quality control filters in the gnomAD genome datasets and is only visible when the ‘filtered variants’ checkbox is selected. In contrast, it is a PASS variant in the exome subset of gnomAD v2.1.1. This inconsistent AF data likely explains the conflicting interpretations in ClinVar—currently one benign, one likely benign, two likely pathogenic and two pathogenic assessments. This degree of conflict is unusual, as diagnostic laboratories apply ACMG guidelines conservatively and typically report variants as being of uncertain significance when doubt arises.

**Figure 1:**
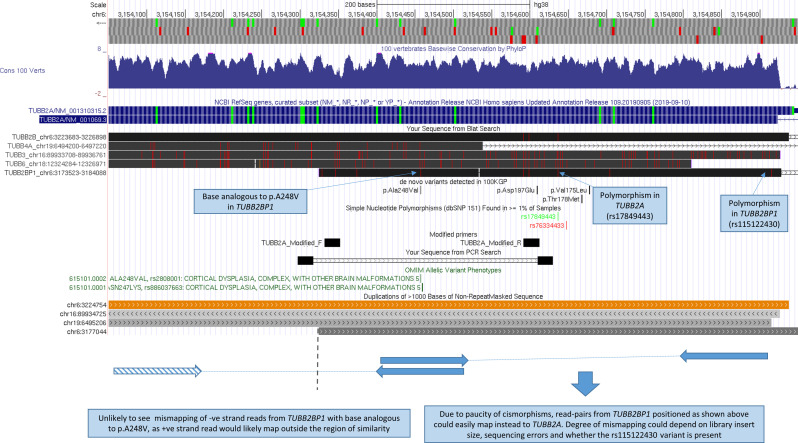
Relative positions of segmental duplications and hypothesis for strand bias associated with NC_000006.12:g.3154458G>A, p.(Ala248Val). Customised UCSC genome browser session highlighting the positions of segmental duplications showing at least 90% identity (interactive version at: https://genome.ucsc.edu/s/AlistairP/TUBB2A_v5). Region shown corresponds to a 900 bp section of exon 4. The RefSeq annotation corresponding to the canonical *TUBB2A* isoform is highlighted. The positions of primers used in the Cushion *et al* study are indicated by the in silico PCR track—the lack of cismorphisms at these sites suggests that *TUBB2A* and *TUBB2B* would both be amplified with an equal efficiency. The position of a modified reverse primer which contains mismatches with *TUBB2B* at the 3′ end is indicated. The position of the base in *TUBB2BP1* that is analogous to p.(Ala248Val) is labelled. Cismorphisms at sites which are also polymorphic in *TUBB2A* or *TUBB2BP1* are also labelled. Other de novo variants detected in 100KGP are indicated, although p.(Val49Met) and p.(Arg391His) are not shown as they lie outside the region shown. The schematic diagram below the UCSC session indicates relative positions of hypothetically mismapped read pairs from *TUBB2BP1* (which lies 23 kb proximal to *TUBB2A*), which could explain the strand-bias observed. Negative strand reads from *TUBB2BP1* that harbour the base analogous to p.(Ala248Val) are unlikely to mismap to *TUBB2A* as the corresponding +ve strand paired read then would lie outside the region of similarity. 100KGP, 100,000 Genomes Project; *TUBB2A*, beta-tubulin isotype 2A.

Segmental duplications are known to result in reads with low mapping quality on short-read sequencing, and this can cause mismapping artefacts. Indeed, several regions share similarity with *TUBB2A*. Although the highest identity is with *TUBB2B*, other beta tubulin genes (*TUBB3/TUBB4A/TUBB6*) and a pseudogene (*TUBB2BP1*) share >90% identity with *TUBB2A* exon 4 (online supplemental table S1). Notably, *TUBB2BP1* contains the analogous base to p.(Ala248Val) in *TUBB2A*, and this ‘cismorphism’ is in a region relatively depleted for other cismorphisms (figure 1). Thus, we speculate that mismapping of reads from *TUBB2BP1* may result in p.(Ala248Val) being called in *TUBB2A* as an artefact and thus the apparently high AF in gnomAD.

10.1136/jmedgenet-2020-107528.supp1 Supplementary data

**Figure 2:**
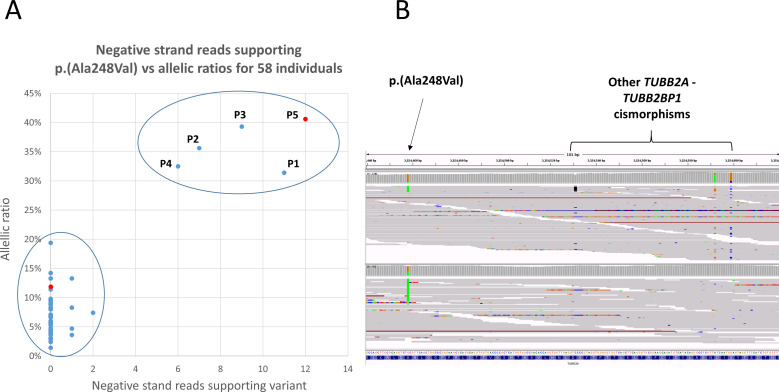
(A) Allelic ratios for p.(Ala248Val) plotted against the number of negative strand reads supporting the variant in 58 individuals from the 100KGP. Five patients have an allelic fraction of >30%, and the variant is also supported by six or more of negative reads. These variants were considered to be real and form a discreet cluster compared with the 53 cases with low allelic fractions which are supported almost exclusively by +ve strand reads. Patients 1–5 are labelled P1–P5. (B) Read alignments shown in the Integrative Genomics Viewer for one case with likely artefact (upper) alongside the likely genuine variant in patient 5 (lower). The samples shown correspond to the red data points shown in panel A. Reads are sorted by base and shown using the squished option. Three other cismorphisms in the same reads are highlighted—the similarly low allelic fractions are consistent with a mismapping artefact. In the lower panel, as well as higher allelic fraction, multiple reads supporting the variant are seen on both strands. 100KGP, 100,000 Genomes Project; *TUBB2A*, beta-tubulin isotype 2A.

Searching data from 78 195 individuals sequenced as part of the 100KGP (online supplemental material, Methods section) uncovered 58 subjects apparently heterozygous for p.(Ala248Val). On reviewing read alignment statistics, two distinct clusters were seen. In 5/58 individuals, the p.(Ala248Val) variant appeared with allelic ratios of 31%–41%, supported by multiple reads across both strands. In contrast, for the remaining 53 individuals, the variant was observed at lower allelic fractions (1%–19%), almost exclusively on positive strand reads (figure 2A). The strand bias is similar to that seen in gnomAD v2.1.1 and could be explained if the variant was a mismapping artefact due to reads from *TUBB2BP1*, as the region of similarity extends distally by only 133 bp (figure 1). This mismapping hypothesis is also supported by three nearby *TUBB2A-TUBB2BP1* cismorphisms, which can be observed in the same reads (figure 2B).

All five patients with apparently ‘genuine’ variants had neurodevelopmental presentations involving intellectual disability. Three patients were reported to have seizures (one with electroencephalogram showing hypsarrhythymia); three had hypoplasia of the corpus callosum; and three had asymmetric ventricules; the findings were not atypical of the clinical tubulinopathy spectrum (online supplemental table S2 and figure S1). In four of five of these cases, genome sequencing had been performed as parent–child trios, and in these, the variant was confirmed to have arisen de novo. The other 53 individuals spanned several disease areas and included unaffected family members, as well as germline samples from patients with cancer (online supplemental table S3).

10.1136/jmedgenet-2020-107528.supp2 Supplementary data

10.1136/jmedgenet-2020-107528.supp3 Supplementary data

Of the five patients where the variant was suspected to be genuine, three were white; one was Pakistani; and for one, ethnicity data were unavailable. Of the remaining 53 individuals, 34% were African/Caribbean; 30% were Asian; 13% were white; and for 23%, ethnicity data were not available. The increased prevalence of likely artefactual variant calls in individuals of African ethnicity mirrors the pattern seen in gnomAD. This may reflect *TUBB2BP1* polymorphisms or additional tracts of common paralogous sequence in that population.

On a technical note, where Sanger sequencing is used for validation, primer design is critically important. In the original study by Cushion *et al*,^1^ a low allelic fraction was observed in the electropherogram. Rather than reflecting mosaicism, this was likely due to coamplification of *TUBB2B* (figure 1). We propose an alternative reverse primer (online supplemental table S4) that increases specificity towards *TUBB2A* and also demonstrate that poor primer design can lead to erroneous validation of NGS artefacts (online supplemental figure S2). Where similar methods are used, we recommend filtering p.(Ala248Val) variant calls at an allelic fraction of >20% and requiring >2 reads on both strands.

For one case, retrospective analysis of exome sequencing validated p.(Ala248Val) but further emphasised the impact of read lengths on mapping quality (online supplemental figure S3). Applying a similar analytical strategy on *TUBB2B* identified two patients from 100KGP with cortical brain malformations harbouring the corresponding p.(Ala248Val) variant (online supplemental material), with a similar clustering pattern observed (online supplemental figures S4, S5).

10.1136/jmedgenet-2020-107528.supp4 Supplementary data

Our cautionary tale highlights the difficulty in distinguishing bona fide gene-conversion events from mapping artefacts using short-read data. It is anticipated that increased uptake of long-read sequencing technologies will be beneficial to help fully resolve repetitive loci such as this.^10^ The value of plotting read-alignment statistics across a large cohort of individuals analysed using a uniform pipeline (eg, 100KGP) is also highlighted. It is likely that similar approaches may be useful for other genes where conversion events represent an important mutational mechanism.
