# Supplementary material for: Using data from the 100,000 Genomes Project to resolve conflicting interpretations of a recurrent TUBB2A mutation
Source: J Med Genet. 2021 Feb 5;59(4):366–9. doi: 10.1136/jmedgenet-2020-107528 (PMC8961759; doi:10.1136/jmedgenet-2020-107528)
Supplement: Supplementary data [file jmedgenet-2020-107528supp003.pdf]

Using data from the 100,000 Genomes Project to resolve conflicting interpretations of a recurrent *TUBB2A* mutation

Online supplemental material

Supplemental methods

The 100K Genomes Project (100KGP) is a national genome sequencing initiative approved by the HRA Committee East of England, Cambridge South (REC: 14/EE/1112). More information about this project is available online (www.genomicsengland.co.uk and https://doi.org/10.6084/m9.figshare.4530893.v5). Germline DNA samples from 78,195 individuals were sequenced using a 150bp paired-end format in a single lane of an Illumina HiSeq X instrument. Reads were aligned to the GRCh38 assembly (with decoys) using the iSAAC Aligner v03.16.02.19 and small variants were called using Starling v2.4.7. Aggregation of single-sample gVCFs were performed using gVCF genotyper v2019.02.29 (Illumina) and normalisation/decomposition was implemented by vt version 0.57721. The multisample VCF was split into 1,371 roughly equal chunks to allow faster processing and the loci of interest were queried using bcftools v1.9.

In order to validate the p.(Val248Ala) variants in *TUBB2A* and *TUBB2B*, PCR reactions were performed in 25µl volumes using the Megamix (Microzone) or FastStart (Roche) kits along with the primers listed in Table S4 Thermocycling conditions included 5 minutes of initial denaturation at 95°C, followed by 30 cycles of 1 minute at 95°C, 1 minute at 58°C and 1 minute at 72°C. A final extension for 10 minutes was performed at 72°C. Prior to sequencing, PCRs reactions were purified using Exonuclease I and Shrimp Alkaline Phosphatase. Sequencing reactions were performed using BigDye v3.1 chemistry and samples were run on an ABI 3730xl/3130xl instrument.

Supplemental discussion

Incidence and mechanism underlying recurrent p.(Ala248Val) mutations

In this study we identified 5 cases with the likely genuine p.(Ala248Val) mutation, of which 4 were confirmed as having arisen *de novo*. This recurrent mutation was identified by searching through 78,195 study participants, of whom 6,570 had been recruited to the 100KGP with a neurodevelopmental disorder. All 5 mutation carriers were in the latter category - we did not identify any likely genuine p.(Ala248Val) mutations amongst the 71,625 participants recruited for a different reason. As well as giving an idea of the likely incidence of this mutation in cohorts of individuals with neurodevelopmental disorders, this bias (5/6,570 vs 0/71,625; $P < 0.00001$, Fisher's exact test) also helps strengthen the evidence supporting the pathogenicity of p.(Ala248Val). In contrast, only 4/53 cases with the likely artefactual variant call were from the neurodevelopmental subdomain and this distribution was consistent with the null hypothesis (4/6,570 vs 49/71,625; $P > 0.05$).

Deamination of methylated cytosine residues is the most common mutational mechanism and such variants are approaching saturation in large population genome datasets.[1] Although the p.(Ala248Val) mutation involves a C>T transition, it is not at a CpG dinucleotide and so the recurrence is unlikely to result from this

mutational process. We suspect that, as well as resulting in missmapping artefacts, the cismorphic base in *TUBB2BP1* might explain why genuine p.(Ala248Val) variants in *TUBB2A* are recurrent, via gene conversion. Gene-conversion events are increasingly being recognised as an important mutational mechanism.[2] Well known examples include recurrent mutations in *SBDS*,[3] *GBA*[4 5] and the c.757delG variant in *SORD* recently associated with hereditary neuropathy.[6] The likelihood of gene conversion events occurring is correlated with how far the region of sequence similarity is away from the target site[2]; one study suggested that most conversion events occur where the duplication is <55kb away.[7] In the case of *TUBB2A, TUBB2BP1* lies approximately 23kb away and so well within the range where conversion events become more common.

Detection of genuine mosaicism

It is well known that parent-child trio sequencing using NGS is an effective way of picking up genetic mosaicism.[8] However in this case, mosaicism of the p.(Ala248Val) variant would be very difficult to detect robustly given our recommendation to use an allelic ratio threshold >20% to remove artefactual variant calls.

Reasons why p.(Ala248Val) was missed previously

We note that p.(Ala248Val) was not reported by the clinical filtering pipeline used by the 100KGP likely due to the Platypus 0.8.1 variant caller annotating variant with either a MQ (4/5) or a badReads (1/5) warning flag. In contrast, in the single sample Starling small-variant call vcfs, p.(Ala248Val) was annotated with a PASS flag in 5/5. We also note that the variant is called by GATK and passes the recommended hard filters in 5/5. In Patient 4, the variant was missed by the DDD study's filtering pipeline because PolyPhen predicts p.(Ala248Val) to be benign and this rules the variant out for analysis of singleton datasets (pers. comm. Caroline Wright), even though this gene has a high missense constraint score (Z=5.26; gnomAD v2.1.1).

Searching for p.(Ala248Val) in *TUBB2B* in 100KGP

It is notable that the analogous variant p.(Ala248Val) in *TUBB2B* has also been described in the literature in patients with polymicrogyria, intellectual disability and epilepsy.[9] A different variant involving the same codon p.(Ala248Thr) has also been reported previously as a *de novo* variant in a 28.5 week old foetus with central polymicrogyria-like cortical dysplasia and mild vermis hypoplasia.[10]

The p.(Ala248Val) *TUBB2B* variant is associated with similarly conflicting interpretations in ClinVar (www.ncbi.nlm.nih.gov/clinvar/variation/381699); currently 1 benign and 1 likely pathogenic. The variant is a filtered (low-confidence) variant in gnomAD v2.1.1 and is present with a slightly higher allele frequency (4,284/134,020) in the exomes compared to the genomes (441/20,198). In gnomAD v3.1 the variant is also filtered and the global allele frequency of 1,900/120,372 rises to 1,668/23,178 (7.2%) in African/African Americans. In theory, this high AD would meet the BA1 stand-alone criteria supporting a benign interpretation using the ACMG variant interpretation framework.[11] However the lack of homozygotes (>50 expected in African/African Americans in gnomAD v3.1 assuming Hardy-Weinburg equilibrium; none observed) and a review of read alignments available for gnomAD v3.1 showing strand bias (only a single -ve strand read supporting the variant, across 20 heterozygous genomes available at time of review) strongly suggested this "variant" to be artefactual in most cases.

TUBB2B lies further away from *TUBB2BP1* than *TUBB2A*, so genuine gene conversion events might be expected to be less common. However *TUBB2B* and *TUBB2BP1* are still within the <55kb range proposed by Ezawa *et al* where the majority of conversion events occur.[7] We therefore sought to replicate the results seen for *TUBB2A* and searched for the analogous position in *TUBB2B,* as described above. Of the 78,195 individuals from the 100KGP contained in the V2 aggregate vcf file, we identified 7 individuals apparently heterozygous for p.(Ala248Val). In 3/7 of these individuals, the p.(Ala248Val) variant appeared with allelic ratios of >20% and supported by multiple reads across both strands. In contrast, for the remaining 4 individuals, the variant was observed at lower allelic fractions (3.5-13.5%) and was almost exclusively supported by +ve strand reads (Figure S4A). The clustering pattern seen is strikingly similar to that presented for *TUBB2A* in Figure 2A.

Of the three individuals with plausible p.(Ala248Val) variants in *TUBB2B,* one was a case where limited clinical information was available. For this individual, the variant was seen at an allelic fraction of 24% and supported by four -ve strand reads; however upon close scrutiny, there was an additional nearby cismorphism (delC) seen in overlapping reads (Figure S4B), raising the possibility that a subset of reads could originate from *TUBB2BP1*. The other two individuals (allelic fractions >20%, six -ve strand reads supporting the variant) were recruited to the 100KGP under the diagnosis category "Malformations of cortical development". Of the samples contained in the aggregate vcf, only 153/78,195 (i.e. <1/500) were from individuals entered into the 100KGP under that specific disease category. Given that *TUBB2B* is linked to a single condition in OMIM "Cortical dysplasia, complex, with other brain malformations 7" (MIM #615763), finding cases in this category would have been the *a priori* expectation. The chances of both cases with the most plausible p.(Ala248Val) variants falling into this category would have been <1/250,000 under the null hypothesis. Unfortunately, neither of these patients were recruited to the 100KGP as part of parent-child trios and so we cannot determine whether these variants may have occurred *de novo.* Sanger sequencing was used to validate the variant for one of the two cortical malformation cases, where DNA was available (Figure S5).

Lastly, we note that *TUBB2B* is better established as a disease gene and the associated cortical phenotypes are generally more profound than those seen in *TUBB2A* patients.[10 12] The fact that all five *TUBB2A* positive cases were entered into the 100KGP with a diagnosis of intellectual disability whereas the two suspected *TUBB2B* cases were listed in the cortical malformation disease category is therefore consistent with the literature and highlights that for the latter condition, MRI imaging typically plays a more prominent role in the diagnostic workup.

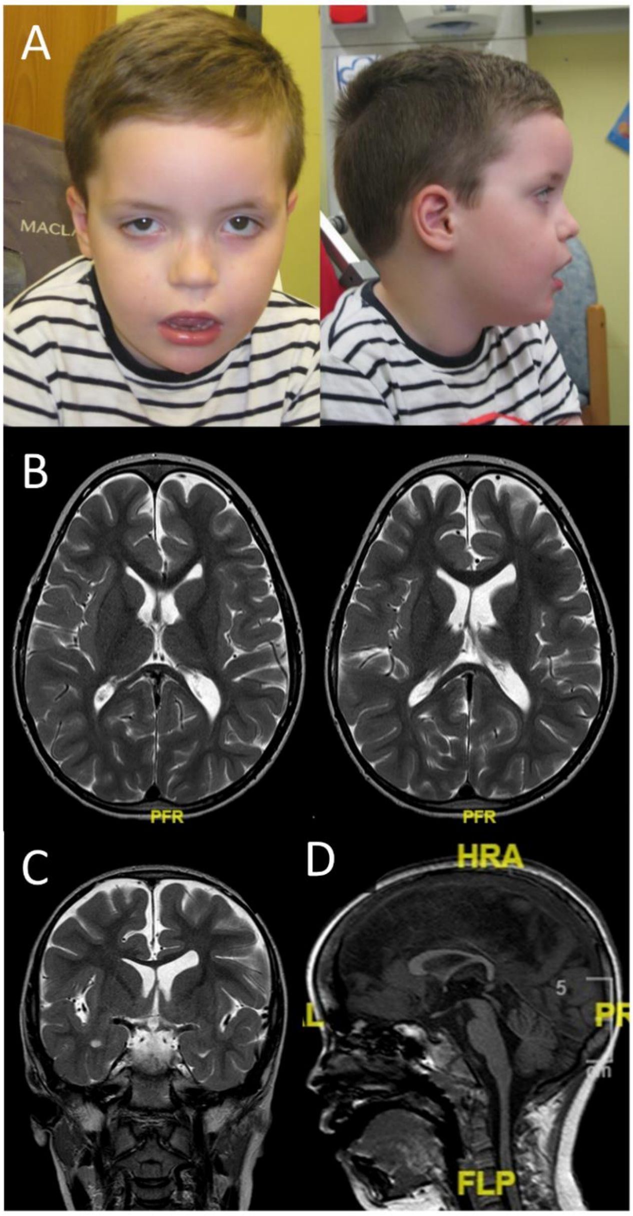

Figure S1: Photographs and MRIs for Patient 1 obtained with parental consent. A) Facial photographs showing frontal bossing, laterally sparse eyebrows, mild hypertelorism, proptosis, depressed nasal bridge, long philtrum, thin tented upper lip, everted lower lip and drooling. B) Axial T2W MRI images showing oversimplification of gyral pattern of frontal cortex with dysgyric cingulate cortex anteriorly, asymmetry of lateral ventricles, and normal basal ganglia. C) Coronal T2W MRI image showing oversimplification of gyral pattern of anteriorcortex with dysgyric cingulate cortex anteriorly, asymmetry of lateral ventricles, and normal basal ganglia. There is no evidence of polymicrogyria. D) The midline sagittal T1W image shows thin corpus callosum and a degree of inferior vermis hypoplasia. Clinical images are not available for Patients 2-5.

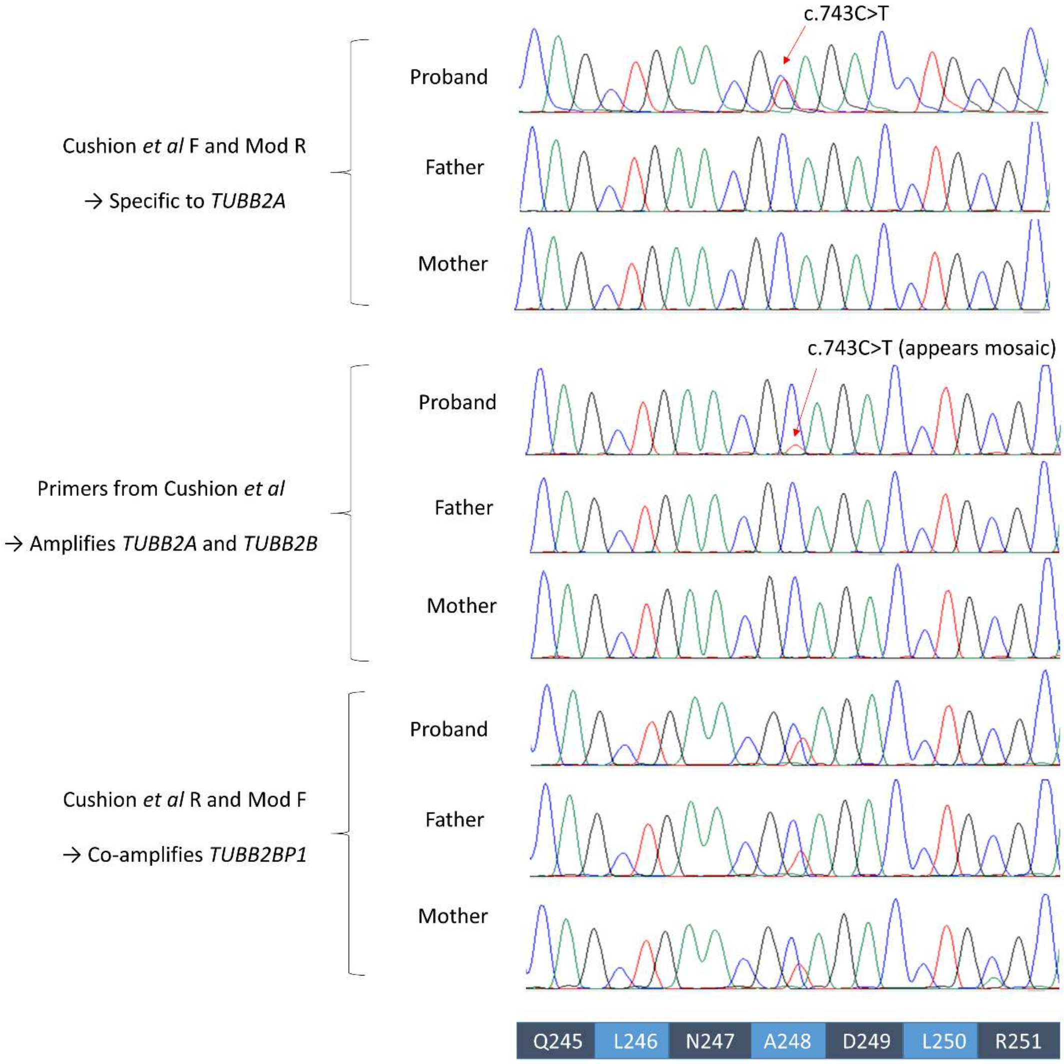

Figure S2: Sanger validation results for the p.(Ala248Val) *TUBB2A* variant in Patient 1 and his parents. Primers used by Cushion *et al*[13] are likely to co-amplify *TUBB2B* and so result in the false impression of mosaicism (middle panel). Using a modified R primer (upper panel) leads to higher specificity towards *TUBB2A*. In contrast, a modified F primer (lower panel) leads to co-amplification of *TUBB2BP1*, which could lead to falsely validating a NGS mapping artefact. The minor peak for the A allele at the first position of codon 251 in the mother corresponds to a polymorphism in *TUBB2BP1* (rs3734485). Similar results were obtained for Patient 4 (data not shown).

Ragoussis V, *et al. J Med Genet* 2022; 59:366–369. doi: 10.1136/jmedgenet-2020-107528

TUBB2A variant interpretation

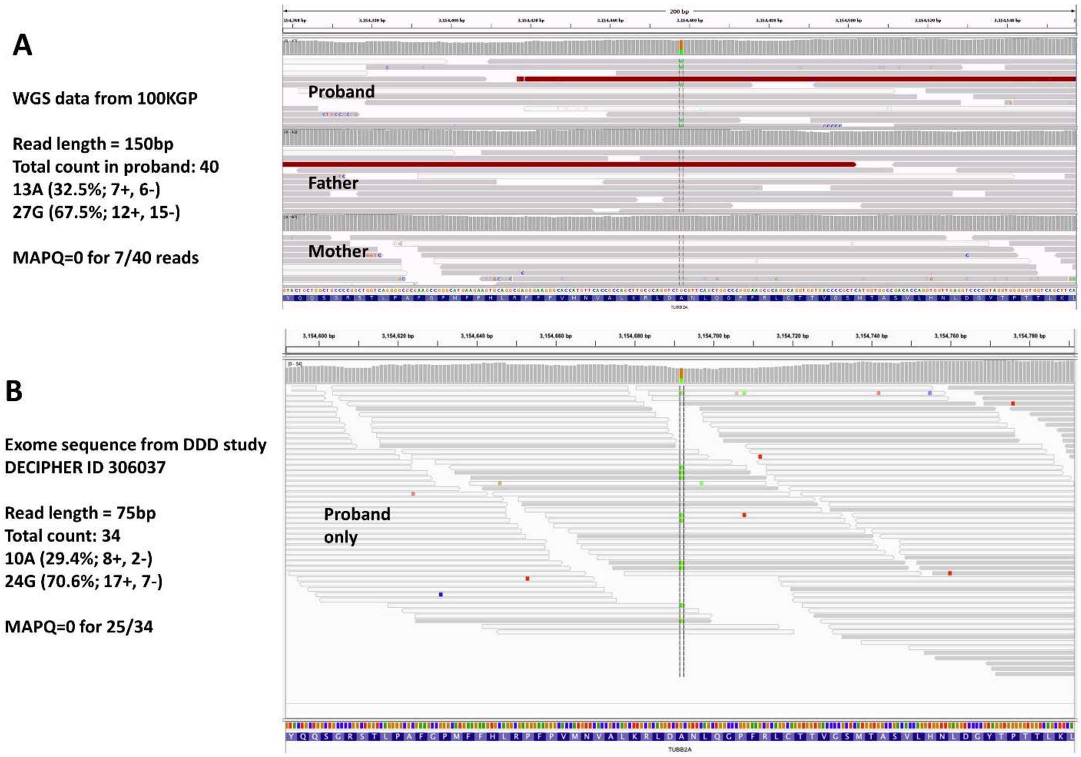

Figure S3: Comparison of genome and exome sequences for Patient 4 who had previously also been enrolled into the DDD study as a singleton (DECIPHER 306037). A lower proportion of MAPQ=0 (white) reads is observed with 150bp read genome sequencing data (A) compared to 75bp read exome sequencing data (B). In both data sets, the p.(Ala248Val) variant is supported by reads on both strands. In the 100KGP, the parent's father and mother were also sequenced and the A allele was observed in 0/42 and 0/59 reads, respectively.

Ragoussis V, *et al. J Med Genet* 2022; 59:366–369. doi: 10.1136/jmedgenet-2020-107528

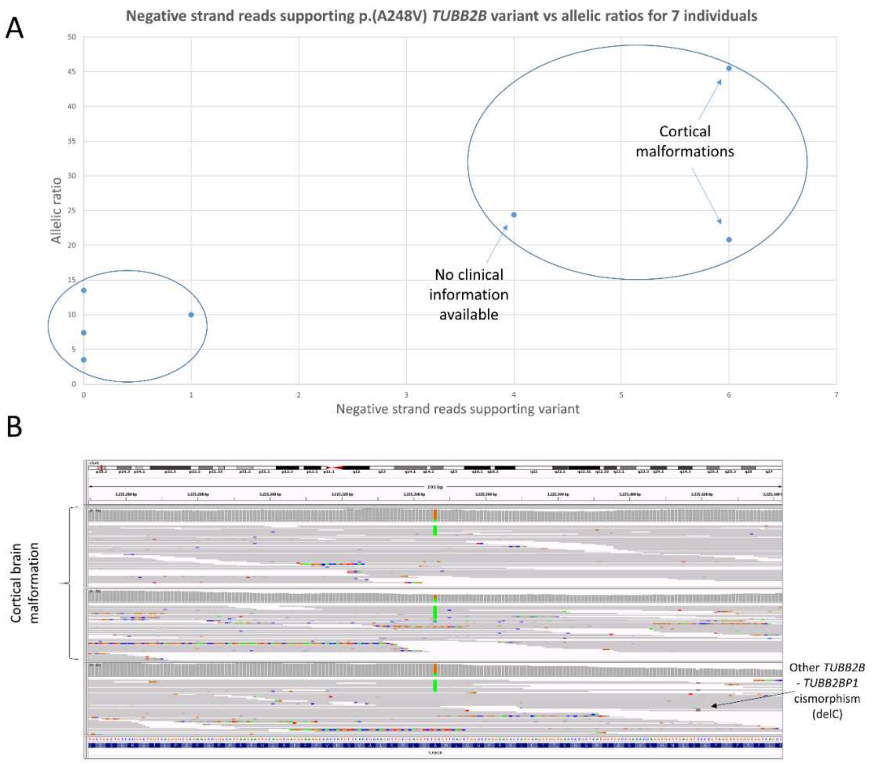

Figure S4: Replication of the read alignment analysis and clustering for individuals with the same p.(Ala248Val) variant in the closely paralogous *TUBB2B* gene. A) Allelic ratios for p.(Ala248Val) plotted against the number of negative strand reads supporting the variant in 7 individuals from the 100KGP. Three patients have an allelic fraction >20% and the variant is also supported by 4 or more of negative reads. These variants appear to form a discreet cluster compared to the 4 cases with low allelic fractions which are supported almost exclusively by +ve strand reads, replicating the pattern seen for *TUBB2A* in Figure 2A. B) Read alignments shown in IGV for cases with plausible p.Ala248Val variants. Reads are sorted by base and shown using the squished option. In the upper two panels, allelic fractions of >20% are observed and the variant is also supported multiple reads on both strands. Both datasets come from patients recruited to the 100KGP with a cortical brain malformation. In the lower panel, a delC cismorphism is highlighted in overlapping reads - the similarly low allelic fractions are consistent with a mismapping artefact.

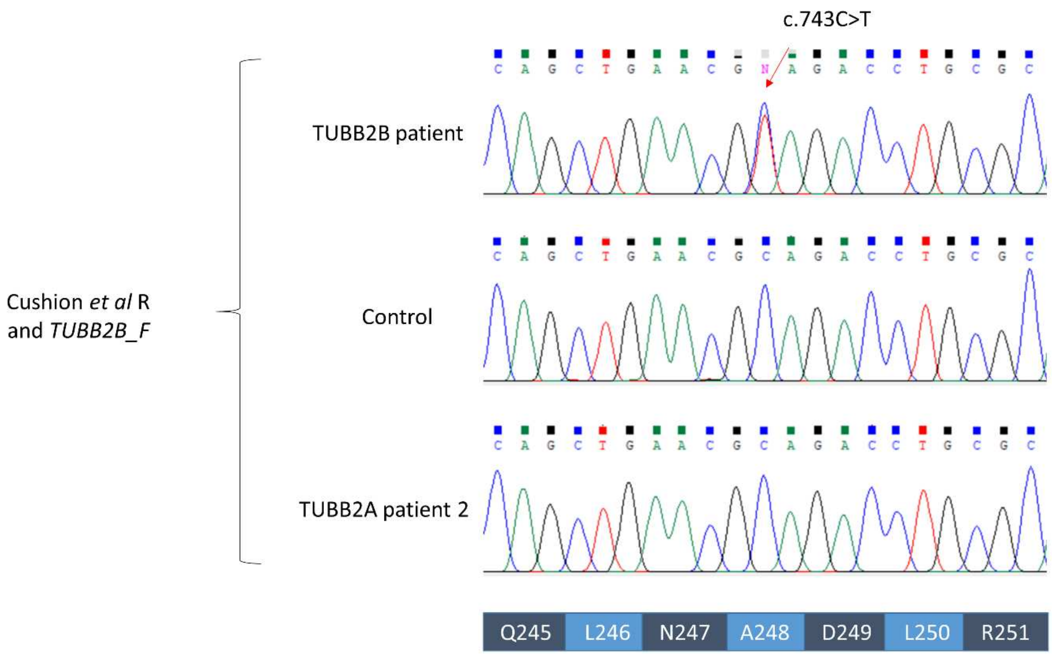

Figure S5: Sanger validation results for the p.(Ala248Val) *TUBB2B* variant in a patient with cortical brain malformations. In the Illumina genome sequencing data, the variant in this patient had been seen with a 21% allelic fraction, as shown in Figure S4. The universal reverse primer used by Cushion *et al*[13] was used together with the TUBB2B_F primer listed in Table S4. No evidence of the mutation was seen in the sample from *TUBB2A* Patient 2, confirming that the F primer results in specificity for the *TUBB2B* isoform.

Table S1: DNA and amino-acid identity for TUBB2A compared to other members of the *TUBB* gene family. *See xlsx file submitted as a separate document.*

Table S2: Clinical and imaging features of patients with variant c.743C>T; p.(Ala248Val) in *TUBB2A*. *See xlsx file submitted as a separate document.*

Table S3: Diagnostic categories for individuals with low confidence p.(Ala248Val) variant in *TUBB2A*.

Unaffected or not available	19
Cancer	9
Intellectual disability	4
Early onset/familial intestinal pseudo obstruction	3
Kidney disease (rhabdomyolysis, cystic kidney disease, proteinuria renal disease)	3
Heart disease/heart problems (dilated cardiomyopathy)	2
Encephalopathy	2
Inherited macular dystrophy	2
Hypertension	2
Congenital hearing impairment	1
Charcot-Marie-Tooth disease	1
Hereditary ataxia	1
Multiple epiphyseal dysplasia	1
Epilepsy	1
Osteogenesis Imperfecta Osteopenia	1
Brain channelopathy	1
Total	53

Table S4: Primer sequences for Sanger validation. Primers labelled Cushion_F and Cushion_R are described in Cushion et al 2014.[13] The binding sites of all 4 *TUBB2A* primers are shown in Figure 1. In contrast to other studies which have used long-range PCR to increase specificity to a target gene of interest,[5 14] here we designed Modified_R to contain two 3' mismatches with *TUBB2B* (underlined bases) so as to increase specificity towards *TUBB2A*. In contrast, Modified_F was designed within the sequence similar to *TUBB2BP1* to test whether this would replicate the artefactual p.(Ala248Val) result. *We note that the presence of a rare variant in *TUBB2B* (rs1054332; 0.48% in gnomAD v3.1) means that in a small fraction of cases there would be just a single mismatch.

**Primer Name**	**Primer sequence**
TUBB2A_Cushion_F	GGCCATCATGTTCTTGGAGT
TUBB2A_Cushion_R	CAGCTGGTGGAAAACACAGA
TUBB2A_Modified_F	TGAGCTCGGGCACCGTGAGC
TUBB2A_Modified_R*	GATGAAACCTACT<u>C</u>CATTGA<u>T</u>
TUBB2B_F	CTCCTCCGCTTTCCCTAACC

Supplemental References

1. Karczewski KJ, Francioli LC, Tiao G, et al. The mutational constraint spectrum quantified from variation in 141,456 humans. *Nature* 2020;581(7809):434-43. doi: 10.1038/s41586-020-2308-7 [published Online First: 2020/05/29]
2. Chen JM, Cooper DN, Chuzhanova N, et al. Gene conversion: mechanisms, evolution and human disease. *Nat Rev Genet* 2007;8(10):762-75. doi: 10.1038/nrg2193 [published Online First: 2007/09/12]
3. Boocock GR, Morrison JA, Popovic M, et al. Mutations in SBDS are associated with Shwachman-Diamond syndrome. *Nat Genet* 2003;33(1):97-101. doi: 10.1038/ng1062 [published Online First: 2002/12/24]
4. Tayebi N, Stubblefield BK, Park JK, et al. Reciprocal and nonreciprocal recombination at the glucocerebrosidase gene region: implications for complexity in Gaucher disease. *Am J Hum Genet* 2003;72(3):519-34. doi: 10.1086/367850 [published Online First: 2003/02/15]
5. Jeong SY, Kim SJ, Yang JA, et al. Identification of a novel recombinant mutation in Korean patients with Gaucher disease using a long-range PCR approach. *J Hum Genet* 2011;56(6):469-71. doi: 10.1038/jhg.2011.37 [published Online First: 2011/04/15]
6. Cortese A, Zhu Y, Rebelo AP, et al. Biallelic mutations in SORD cause a common and potentially treatable hereditary neuropathy with implications for diabetes. *Nat Genet* 2020;52(5):473-81. doi: 10.1038/s41588-020-0615-4 [published Online First: 2020/05/06]
7. Ezawa K, S OO, Saitou N, et al. Proceedings of the SMBE Tri-National Young Investigators' Workshop 2005. Genome-wide search of gene conversions in duplicated genes of mouse and rat. *Mol Biol Evol* 2006;23(5):927-40. doi: 10.1093/molbev/msj093 [published Online First: 2006/01/13]
8. Pagnamenta AT, Lise S, Harrison V, et al. Exome sequencing can detect pathogenic mosaic mutations present at low allele frequencies. *J Hum Genet* 2012;57(1):70-2. doi: 10.1038/jhg.2011.128 [published Online First: 2011/12/02]

9. Amrom D, Tanyalcin I, Verhelst H, et al. Polymicrogyria with dysmorphic basal ganglia? Think tubulin! *Clin Genet* 2014;85(2):178-83. doi: 10.1111/cge.12141 [published Online First: 2013/03/19]
10. Bahi-Buisson N, Poirier K, Fourniol F, et al. The wide spectrum of tubulinopathies: what are the key features for the diagnosis? *Brain* 2014;137(Pt 6):1676-700. doi: 10.1093/brain/awu082 [published Online First: 2014/05/27]
11. Richards S, Aziz N, Bale S, et al. Standards and guidelines for the interpretation of sequence variants: a joint consensus recommendation of the American College of Medical Genetics and Genomics and the Association for Molecular Pathology. *Genet Med* 2015;17(5):405-24. doi: 10.1038/gim.2015.30 [published Online First: 2015/03/06]
12. Brock S, Vanderhasselt T, Vermaning S, et al. Defining the phenotypical spectrum associated with variants in TUBB2A. *J Med Genet* 2020 doi: 10.1136/jmedgenet-2019-106740 [published Online First: 2020/06/24]
13. Cushion TD, Paciorkowski AR, Pilz DT, et al. De novo mutations in the beta-tubulin gene TUBB2A cause simplified gyral patterning and infantile-onset epilepsy. *Am J Hum Genet* 2014;94(4):634-41. doi: 10.1016/j.ajhg.2014.03.009 [published Online First: 2014/04/08]
14. Wang H, Li S, Li S, et al. De Novo Mutated TUBB2B Associated Pachygyria Diagnosed by Medical Exome Sequencing and Long-Range PCR. *Fetal Pediatr Pathol* 2019;38(1):63-71. doi: 10.1080/15513815.2018.1538273 [published Online First: 2018/12/27]
