# Supplementary material for: Using data from the 100,000 Genomes Project to resolve conflicting interpretations of a recurrent TUBB2A mutation
Source: J Med Genet. 2021 Feb 5;59(4):366–9. doi: 10.1136/jmedgenet-2020-107528 (PMC8961759; doi:10.1136/jmedgenet-2020-107528)
Supplement: Supplementary data [file jmedgenet-2020-107528supp004.pdf]

Appendix: The Genomics England Research Consortium*

Ambrose J. C.[1], Arumugam P.[1], Baple E. L.[1], Bleda M.[1], Boardman-Pretty F.[1,2], Boissiere J. M.[1], Boustred C. R.[1], Brittain H.[1], Caulfield M. J.[1,2], Chan G. C.[1], Craig C. E. H.[1], Daugherty L.C.[1], de Burca A.[1], Devereau, A.[1], Elgar G.[1,2], Foulger R. E.[1], Fowler T.[1], Furió-Tarí P.[1], Hackett J. M.[1], Halai D.[1], Hamblin A.[1], Henderson S.[1,2], Holman J. E.[1], Hubbard T. J. P.[1], Ibáñez K.[1,2], Jackson R.[1], Jones L. J.[1,2], Kasperaviciute D.[1,2], Kayikci M.[1], Kousathanas A.[1], Lahnstein L.[1], Lawson K.[1], Leigh S. E. A.[1], Leong I. U. S.[1], Lopez F. J.[1], Maleady-Crowe F.[1], Mason J.[1], McDonagh E.M.[1,2], Moutsianas L.[1,2], Mueller M.[1,2], Murugaesu N.[1], Need A. C.[1,2], Odhams C. A.[1], Patch C.[1,2], Pereira M. B.[1], Perez-Gil D.[1], Polychronopoulos D.[1], Pullinger J.[1], Rahim T.[1], Rendon A.[1], Riesgo-Ferreiro P.[1], Rogers T.[1], Ryten M.[1], Savage K.[1], Sawant K.[1], Scott R. H.[1], Siddiq A.[1], Sieghart A.[1], Smedley D.[1,2], Smith K. R.[1,2], Smith S. C[1], Sosinsky A.[1,2], Spooner W.[1], Stevens H. E.[1], Stuckey A.[1], Sultana R.[1], Thomas E. R. A.[1,2], Thompson S. R.[1], Tregidgo C.[1], Tucci A.[1,2], Walsh E.[1], Watters, S. A.[1], Welland M. J.[1], Williams E.[1], Witkowska K.[1,2], Wood S. M.[1,2], Zarowiecki M.[1]

1. Genomics England, London, UK. 2. William Harvey Research Institute, Queen Mary University of London, London, EC1M 6BQ, UK
